# Atmospheric Environment Vulnerability Cause Analysis for the Beijing-Tianjin-Hebei Metropolitan Region

**DOI:** 10.3390/ijerph15010128

**Published:** 2018-01-13

**Authors:** Yang Zhang, Jing Shen, Yu Li

**Affiliations:** Resources and Environmental Research Academy, North China Electric Power University, Beijing 102206, China; rick@ncepu.edu.cn (Y.Z.); 1132102043@ncepu.edu.cn (J.S.)

**Keywords:** atmospheric environment, quantitative vulnerability assessment, Exposure-Sensitivity-Adaptive Capacity framework, fuzzy theory, environmental management

## Abstract

Assessing and quantifying atmospheric vulnerability is a key issue in urban environmental protection and management. This paper integrated the Analytical hierarchy process (AHP), fuzzy synthesis evaluation and Geographic Information System (GIS) spatial analysis into an Exposure-Sensitivity-Adaptive capacity (ESA) framework to quantitatively assess atmospheric environment vulnerability in the Beijing-Tianjin-Hebei (BTH) region with spatial and temporal comparisons. The elaboration of the relationships between atmospheric environment vulnerability and indices of exposure, sensitivity, and adaptive capacity supports enable analysis of the atmospheric environment vulnerability. Our findings indicate that the atmospheric environment vulnerability of 13 cities in the BTH region exhibits obvious spatial heterogeneity, which is caused by regional diversity in exposure, sensitivity, and adaptive capacity indices. The results of atmospheric environment vulnerability assessment and the cause analysis can provide guidance to pick out key control regions and recognize vulnerable indicators for study sites. The framework developed in this paper can also be replicated at different spatial and temporal scales using context-specific datasets to support environmental management.

## 1. Introduction

With the booming industrialization and urbanization development, the relationship between environmental quality and human society has come into obvious disharmony [1,2]. The continuous deterioration of atmospheric quality in urban cities, especially in developing countries, is one of the top global crises, engendering severe human health, environmental, social, and economic impacts [3,4]. One of the major future challenges will be to restrain short- and long-term atmospheric environment degradation to fulfil the needs of the fast-growing human population and changes in climate, where effective management and conservation have a key role. In this context, research on efficient recognition of dominant influencing factors and vulnerable areas according to specific circumstance is urgently needed. As an effective means of characterization and identification of the extent to which a system is susceptible to damage from natural and anthropogenic disturbance, atmospheric environment vulnerability assessment is an effective solution to this issue.

To date, most studies about vulnerability assessment have focused on urban groundwater contamination [5,6], climate change [7,8], human ecology [9,10], agriculture [11,12], coastal flooding [13], soil [14,15], socioeconomic issues [16,17], wetland biota [18], transportation networks [19], etc. Berrouet et al. proposed a conceptual framework for the vulnerability assessment of socio-ecological systems to communicate with practitioners and policy makers and identify and improve the factors that reduce vulnerability [9]. The results showed that the developed framework can serve as a tool for targeting the implementation of policies and practices aimed at reducing vulnerability. Klaas et al. presented a Head-Guided Zonation (HGZ) method to develop groundwater vulnerability zones in Rote Island, Indonesia, which is under potential risk of contamination from rapid land use changes [6]. The simulation results were combined with the potential groundwater contamination risk from human activities to develop three vulnerability zones. The corresponding preventive management strategies were proposed to protect the spring from contamination. However, there are very few studies that consider atmospheric environment vulnerability as a research subject [20]. It is necessary to build a comprehensive and robust atmospheric environment vulnerability assessment framework to identify vulnerable areas with dominant influencing factors.

The developed atmospheric environment vulnerability assessment framework is demonstrated to be valid in districts where natural and anthropogenic indices are effective at measuring current atmospheric environment vulnerability. Taking the time dimension into account makes this framework able to evaluate the influence of historical policies on atmospheric environment vulnerability and provide guidance for further atmospheric environment vulnerability management. The proposed framework allows for a comparison of atmospheric environment vulnerability if the status quo is maintained or if new decisions are introduced and allows for a representation of natural and anthropogenic change under intrinsic or extrinsic stresses.

## 2. Methods 

### 2.1. Atmospheric Environment Vulnerability Assessment Framework

The concept of atmospheric environment vulnerability can be defined as: “The degree to which a system is susceptible to, or unable to cope with, adverse effects of climate change, including climate variability and extremes. Vulnerability is a function of the character, magnitude and rate of climate variation to which a system is exposed, its sensitivity and its adaptive capacity [21]”.

Numerous approaches have been proposed to evaluate vulnerability: the Exposure-Sensitivity-Adaptive Capacity (ESA) Framework [21,22,23], the Pressure-Support-State-Response (PSSR) model [24], the Driver-Pressure-State-Impact-Response Framework (DPSIR) [25], and the Composite Index (CI) method [26,27]. For now, there is no internationally recognized standard or rule to stipulate how many and what parameters should be selected to capture atmospheric environment vulnerability. Atmospheric environment vulnerability assessment is a complex process. After comparing the other approaches, we recommend the ESA framework to establish the atmospheric environment vulnerability index. It is selected for the following reasons: firstly, the ESA framework can assemble the primary elements of social-atmospheric systems at multiple scales; secondly, it emphasizes adaptive capacity, which shapes vulnerability to a large extent in the long period of atmospheric environment management; last, it uses a combination of different methodologies and indices for a more integrated assessment [28,29].

**Figure 1:**
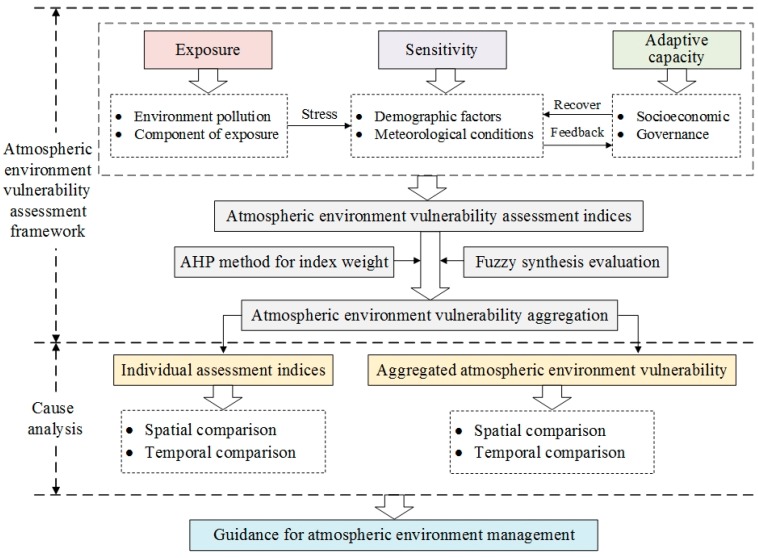
The technical route of atmospheric environment vulnerability assessment.

The concept of ESA framework was proposed by the Intergovernmental Panel on Climate Change [21]. This framework is established from three indices: exposure, sensitivity, and adaptive capacity. The exposure index for atmospheric environment vulnerability assessment is defined as the exposure degree of atmospheric environment to perturbations or stressors. The sensitivity index characterizes the sensitivity of the atmospheric environment or exposure population to perturbations, which refers to parameters that make the atmospheric environment or exposure population vulnerable and easily affected by external disturbance. The adaptive capacity index reflects the ability of the atmospheric environment to cope, i.e., accommodate environmental hazards or policy changes [30]. Considering expert opinion, literature investigations [20,31,32], data availability and local concrete conditions, 15 parameters were chosen to assess atmospheric environment vulnerability in this paper, as shown in Table 1. The exposure index is positively correlated with atmospheric environment vulnerability, meaning higher exposure results in increased atmospheric environment vulnerability, and so does the sensitivity index. On the contrary, the adaptive capacity has a negative relationship with atmospheric environment vulnerability, indicating that a higher adaptive capacity leads to lower vulnerability. The spatial change of atmospheric environment vulnerability in this paper can be defined as the change in vulnerability of different cities. The spatial analysis aims to pick out the most stressed area in the study site. The technical route of atmospheric environment vulnerability assessment is presented in Figure 1.

### 2.2. AHP Method for Index Weights

Multi-criteria decision analysis (MCDA) is an advanced tool to support decision-making that takes several different aspects into account at the same time like AHP, ordered weighted averaging (OWA), weighted arithmetic average (WAA), goal programming (GP), weighted geometric average (WGA), technique for order preference by similarity to an ideal solution (TOPSIS), multi-attribute value theory (MAVT), etc. The purpose of MCDA is to assist a decision maker through the decision-making process via explicit formalized modelling. The AHP-based weight method is the MCDA tool selected in this paper.

AHP was developed by Thomas L. Saaty in the 1970s and has been widely used since then [33,34,35,36]. It is particularly suitable for spatial decision analysis with multiple criteria. AHP offers several advantages over more traditional techniques of weight determination. It can turn multi-objective and multi-criteria problems into a single target with multi-level issues. It is not necessary to ensure statistical generalization and the use of pairwise comparisons can reduce the cognitive burden of index importance. AHP builds a hierarchy (ranking) of decision items using comparisons between each pair of indicators, expressed as a matrix. Paired comparisons produce weighting scores that measure how much importance indicators and criteria have. AHP uses the judgments of decision makers to form a decomposition of atmospheric vulnerability into hierarchies with a combination of qualitative and quantitative methods. 

We select 15 indices that are divided into three project layers: exposure, sensitivity, and adaptive capacity, and the three project layers are grouped into a layer for the goal index: atmospheric environment vulnerability. We applied a questionnaire survey to collect the data that are necessary for AHP. The survey was designed to determine the weights of the atmospheric environment vulnerability indices and taken via interview from 10 atmospheric specialists, environment management specialists, and city management experts. The experts were chosen if they (a) have at least 10 years of working/research experience in a related domain, (b) possess sufficient knowledge in atmospheric environment research, and (c) have a relevant educational background. Since it is difficult to gather all experts together for a brainstorming session or free-flowing discussions in a group, we implemented semi-structured interviews to allow more freedom of conversation. The procedure includes:

1. The objective of the interviews: determining the weights for the 15 indices in the atmospheric environment vulnerability assessment.
2. The major questions include:
  (2.1) What is the contribution rank of exposure, sensitivity, and adaptive capacity for atmospheric environment vulnerability?
  (2.2) What is the contribution rank of “average annual concentration of PM_2.5_”, “average annual concentration of PM_10_”, “average annual concentration of SO_2_”, “average annual concentration of NO_2_”, “days of AQI equal to or better than grade II”, “days of AQI equal to and worse than grade V” for exposure?
  (2.3) What is the contribution rank of “population density”, “percentage of vulnerable groups”, “average annual rainfall”, “average annual wind speed”, “average annual relative humidity” for sensitivity?
  (2.4) What is the contribution rank of “proportion of secondary industry”, “motor vehicle population”, “coal consumption”, “percentage of urban greenery coverage” for adaptive capacity?
  (2.5) What is the total contribution rank of the 15 indices for atmospheric environment vulnerability?

After summarizing answers from interviewed experts, the returned data were applied to make pair-wise comparisons and judgement matrix in the AHP process (shown in Table S1). Thus, the contribution of each index to a higher layer is evaluated and the weight of each index in each hierarchy can be achieved (shown in Appendix A). The obtained weights of the 15 indices were then sent to the 10 experts by e-mail to validate the weighting results. The data obtained from the experts were further analysed by manual content analysis. The final weights of the 15 indices are shown in Table 2. All the consistency ratio (CR) values of each matrix are lower than 0.10, which demonstrates the suitability of the calculated weights. The Geometric Consistency Index (GCI) was also used to measure the consistency of the matrices and the results were all less than the threshold determined by Aguarón and Moreno-Jiménez [37,38]. The results verified the consistency achieved from the CR.

### 2.3. Fuzzy Theory for Index Evaluation

The framework relationship between exposure, sensitivity, and adaptive capacity is inexact. Meanwhile, the concept “vulnerability” is fuzzy and not exact; there is no common idea about the exact threshold to distinguish between vulnerable and not vulnerable. For these reasons, it is suitable to use a fuzzy synthesis evaluation method that can handle the uncertainties and aggregate indicators as well as construct a general atmospheric vulnerability index in atmospheric environment vulnerability assessment.

A fuzzy theory is a mathematical theory that values fuzzy set with membership function in the real unit interval [0,1] [39]. A fuzzy set is a pair *x*, *μ_A_* (*x*): *x* ∈ *X*, in which *μ_A_*: *x*→[0,1]. Fuzzy logic operations include fuzzification, combination, and defuzzification. Fuzzification is a process to transfer all input values into fuzzy membership functions. Combination is to execute all applicable rules in the rule base to compute the fuzzy output functions. Defuzzification is to defuzzify the fuzzy output functions to get “crisp” output values (shown in Appendix B).

It is a key step to determine membership function in fuzzy evaluation. We used a truncated trapezoidal membership function and the vulnerability level is divided into a 1–5 scale on which 1 means potential and 5 means highly vulnerable. As the selected vulnerability indices have positive and negative influence, the positive and negative variables are converted to fuzzy sets by means of positive and negative membership functions, respectively. For a positive impact index, its membership functions are calculated by Equations (1)–(3), and the membership functions of a negative impact index are calculated by Equations (4)–(6).

(1) $$\mu_{1}\left(x\right)=\left\{ \begin{array}{cc} 1 & x\leq A_{1}\\ \frac{A_{2}-x}{A_{2}-A_{1}} & A_{1}<x<A_{2}\\ 0 & x\geq A_{2} \end{array}\right.$$

(2) $$\mu_{i}\left(x\right)=\left\{ \begin{array}{cc} 0 & x\leq A_{i-1}\\ \frac{A_{i-1}-x}{A_{i-1}-A_{i}} & A_{i-1}<x\leq A_{i}\\ \frac{A_{i+1}-x}{A_{i+1}-A_{i}} & A_{i}<x<A_{i+1}\\ 0 & x\geq A_{i+1} \end{array}\right.$$

(3) $$\mu_{5}\left(x\right)=\left\{ \begin{array}{ll} 0 & x\leq A_{4}\\ \frac{A_{4}-x}{A_{4}-A_{5}} & A_{4}<x<A_{5}\\ 1 & x\geq A_{5} \end{array}\right.$$

(4) $$\mu_{1}\left(x\right)=\left\{ \begin{array}{cc} 0 & x\leq A_{2}\\ \frac{A_{2}-x}{A_{2}-A_{1}} & A_{2}<x<A_{1}\\ 1 & x\geq A_{1} \end{array}\right.$$

(5) $$\mu_{i}\left(x\right)=\left\{ \begin{array}{cc} 0 & x\leq A_{i+1}\\ \frac{A_{i+1}-x}{A_{i+1}-A_{i}} & A_{i+1}<x\leq A_{i}\\ \frac{A_{i-1}-x}{A_{i-1}-A_{i}} & A_{i}<x<A_{i-1}\\ 0 & x\geq A_{i-1} \end{array}\right.$$

(6) $$\mu_{5}\left(x\right)=\left\{ \begin{array}{cc} 1 & x\leq A_{5}\\ \frac{A_{4}-x}{A_{4}-A_{5}} & A_{5}<x<A_{4}\\ 0 & x\geq A_{4} \end{array}\right.,$$

where *μ_i_ (x)* is the membership of the atmospheric environment vulnerability index at *i_th_* (*i* = 1, 2,…,5) vulnerability class; *A_i_* is the threshold value of the *i_th_* atmospheric environment vulnerability class.

Establishing an appropriate criterion for different atmospheric environment vulnerability levels is essential. As there is no standard to determine the threshold value at each vulnerability class, we built an atmospheric environment vulnerability assessment index system and classification standard (Table 3) according to the relevant evaluation criteria [40,41,42,43]. The fuzzy membership calculation results for Beijing’s atmospheric environment vulnerability in 2015 are shown in Table 4. 

### 2.4. Atmospheric Environment Vulnerability Aggregation

To achieve general atmospheric environment vulnerability results, the first step is to calculate the membership for the three project layers. Taking the exposure layer as an example, for the study site *x*, the memberships for five scales are calculated by Equation (7), as follows:

(7) $$V_{x,E}=\left[W_{E1},\;W_{E2},\;\cdots W_{E6}\right]\left[\begin{array}{ccc} \mu_{1,E_{1}} & \cdots & \mu_{5,E_{1}}\\ \vdots & \mu_{i,E_{m}} & \vdots \\ \mu_{1,E_{6}} & \cdots & \mu_{5,E_{6}} \end{array}\right].$$

With the same method as above, the memberships of five scales for the project layer sensitivity (*V_x,S_*) and adaptive capacity (*V_x,AC_*) can be achieved. The memberships of five scales for the goal layer atmospheric environment vulnerability are obtained to aggregate the memberships of three project layers by Equation (8):

(8) $$V_{x,AEV}=\sum_{i=1}^{3}V_{x,i}.$$

According to the maximum membership principle, the final atmospheric environment vulnerability result is the maximum value among the memberships for five scales.

## 3. Case Study

### 3.1. Study Area

**Figure 2:**
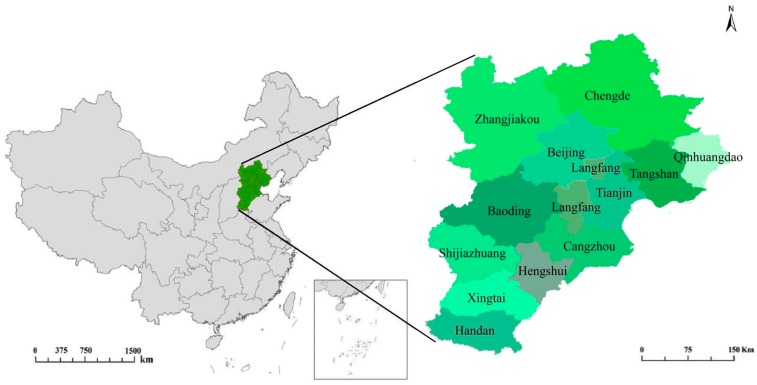
Location and administrative division of the BTH region.

The BTH region (shown in Figure 2) is located in the northwest part of the North China Plain (36°05′–42°37′ N, 113°11′–119°45′ E), which contains two municipalities (Beijing, Tianjin) and one province (Hebei); the total residential population was 111 million at the end of 2015. The total land area of the BTH region is 218,000 km^2^ and occupies 2.3% of the Chinese territory. The BTH region is one of the three hotspot areas of air pollution control according to the Twelfth Five-Year Plan for Air Pollution Control in Key Regions [44]. This area has a semi-humid continental monsoon climate and is in the North Temperate Zone. The Gross Domestic Product (GDP) was 6931.289 billion Chinese Yuan (CNY) in 2015, accounting for 10.24% of the national GDP. According to the China Environmental Status Bulletin in 2015 [51], the air quality of the BTH region was the worst in China, and seven cities in this region ranked in the top 10 for worst air pollution in China.

### 3.2. Data Resource

The following provides the data and sources used in this study. We collected 15 indices of the 13 cities from 2013 to 2015, yielding a total of 585 values. All the input data used in this paper can be divided into three categories: environmental data, meteorological data, and socioeconomic data. The environmental data are derived from the Environment Quality Bulletin [52,53,54,55,56,57,58,59,60], published by Environmental Protection Bureau (EPB) for 13 cities in the BTH region. The meteorological history data of 13 cities are extracted from the China meteorological data sharing service website. Socioeconomic data used in this paper are from the China City Statistical Yearbooks [45,46,47] and the National Economy and Society Developed Statistical Bulletins [48,49,50], which are published by the National Bureau of Statistics of the People’s Republic of China (NBS PRC).

### 3.3. Results Description

**Figure 3:**
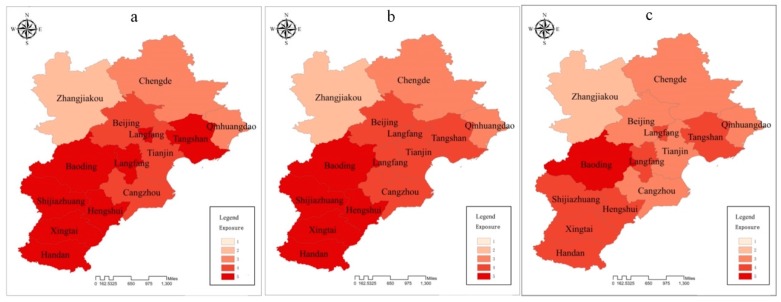
Individual assessment indices of exposure: (**a**) 2013; (**b**) 2014; (**c**) 2015.

**Figure 4:**
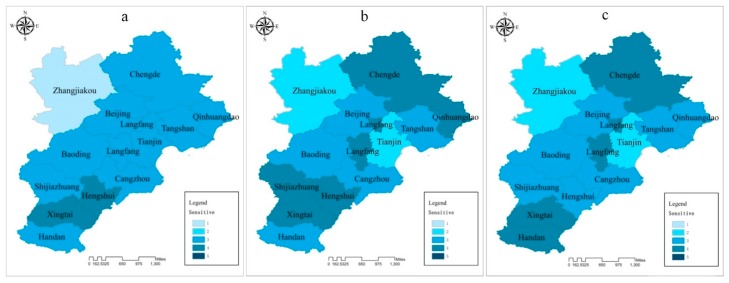
Individual assessment indices of sensitivity (**a**): 2013; (**b**): 2014; (**c**): 2015.

**Figure 5:**
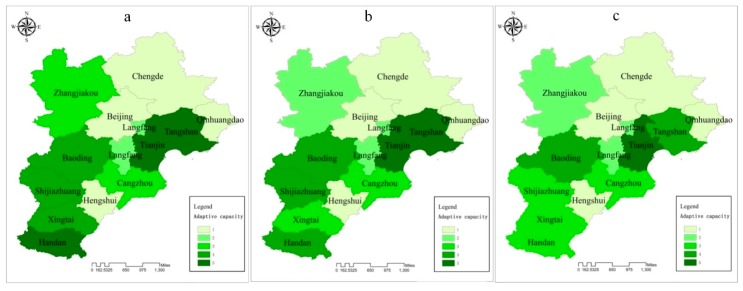
Individual assessment indices of adaptive capacity: (**a**) 2013; (**b**) 2014; (**c**) 2015.

To better depict the spatial and temporal distribution of atmospheric environment vulnerability in BTH region, each assessment index is divided into five levels from 1 to 5, standing for potential vulnerable, weakly vulnerable, moderately vulnerable, strongly vulnerable, and extremely strong vulnerable, respectively. A higher level presents higher impacts on atmospheric environment vulnerability. The assessment results are shown in Figure 3, Figure 4 and Figure 5.

#### 3.3.1. Exposure

Figure 3c indicates that, in terms of exposure index, the fourth-level (46.15% cities of the BTH region) and the third-level (38.46% cities of the BTH region) areas take up the largest proportion of the BTH region in 2015. The exposure index exhibited obvious spatial heterogeneity in the three years. The lowest exposure was mainly found in the northwestern city of Zhangjiakou. Medium exposure dominated in northeastern and central cities; meanwhile, southwestern cities and Tangshan showed the highest exposure. The temporal comparison in the three years showed that the whole exposure situation improved with declining one level in nine cities (Beijing, Tianjin, Shijiazhuang, Tangshan, Handan, Xintai, Cangzhou, Langfang and Hengshui). The three cities (Zhangjiakou, Chengde and Qinhuangdao) showed the lower exposure degree all along, with no change in the three years. Meanwhile, the exposure level of Baoding was always extremely strong and did not change in the three years. 

#### 3.3.2. Sensitivity

Figure 4c shows that the third-level (53.84%) and fourth-level (30.77%) sensitivity indexes account for a major proportion of the BTH region. This suggests that the atmospheric environment of most cities is influenced by perturbations or stressors. The longitudinal comparisons of the three years showed that the sensitivity index also had an obvious spatial discrepancy. The sensitivity degree for Tianjin and Hengshui improved during the research period. On the contrary, the sensitivity levels of Langfang, Handan, Chengde and Zhangjiakou were aggravated.

#### 3.3.3. Adaptive Capacity

Figure 5c shows that the adaptive capacity status in the BTH region is mainly in the first level (30.77%) and third level (30.77%). This indicates that the general adaptive capacity index in the BTH region is relatively positive, with conspicuous spatial heterogeneity generally declining from central cities to southern cities and then to northern cities. The longitudinal comparison from 2013 to 2015 showed that the adaptive capacity situation in the three years presented almost similar patterns in eight cities, while five cites (Shijiazhuang, Tangshan, Handan, Xingtai, and Zhangjiakou) improved. This implies that changes in related adaptive capacity attributes were minor in the three years and half of the cities presented a relatively lower adaptive capacity.

#### 3.3.4. Aggregated Atmospheric Environment Vulnerability

**Figure 6:**
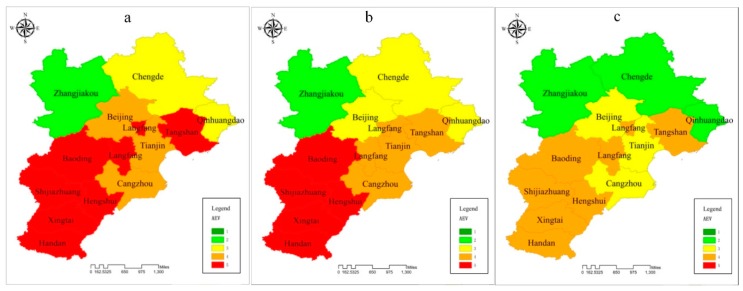
Aggregated atmospheric environment vulnerability assessment: (**a**) 2013; (**b**) 2014; (**c**) 2015.

The assessment results of aggregated atmospheric environment vulnerability are shown in Figure 6. Figure 6c indicates that, in terms of the general atmospheric environment vulnerability, the fourth level (53.84% of 13 cities) took up the largest proportion of the BTH region in 2015. In 2015, the atmospheric environment vulnerability exhibited obvious spatial discrepancy. The general trend shows the vulnerability level increasing from northern to southern regions. In the northern regions, the atmospheric environment in Zhangjiakou, Chengde and Qinhuangdao, at the second level, can be described as stable, with a high capacity to recover from disturbance. In the central regions, Beijing, Tianjin and Cangzhou represented moderately vulnerable atmospheric vulnerability, which indicated the medium anti-interference capacity of the atmospheric system. The widely distributed area with fourth level in the southwestern regions (Langfang, Baoding, Shijiazhuang, Hengshui, Xingtai and Handan) and Tangshan can be treated as an unstable atmospheric environment with a low anti-interference ability for disturbance. This suggests that the atmospheric environment of these cities is more potentially affected by minatory disturbance. For a temporal comparison, the general atmospheric vulnerability of 12 cities improved with declining level and Zhangjiakou remained at a weakly vulnerable level with little change.

## 4. Discussion

The atmospheric environment vulnerability of the BTH region showed diverse patterns in space. There are two patterns in the northern cities (Zhangjiakou, Chengde and Qinhuangdao). The weakly vulnerability of Zhangjiakou was caused by weakly exposure, sensitivity, and adaptive capacity. In the other two cities, the potential adaptive capacity relieved the moderate exposure and sensitivity, resulting in weakly atmospheric environment vulnerability. In the three central cities (Beijing, Tianjin and Cangzhou), the moderate vulnerability was principally influenced by moderate exposure and sensitivity. In the other seven cities with strong vulnerability, there were two possible patterns. One was for Langfang and Hengshui, where the potential and weakly adaptive capacity could not relieve the strong vulnerability to exposure. The other was for Shijiazhuang, Tangshan, Handan, Xingtai and Baoding, showing that the exposure, sensitivity and adaptive capacity were all at moderate to strong vulnerable levels.

The diverse patterns of atmospheric environment vulnerability in space are reasonable. As the sensitivity in most cities was at a high degree with tiny temporal and spatial differences, the spatial change of atmospheric environment vulnerability in the BTH region was mainly influenced by differences in exposure and adaptive capacity indices. In the exposure indices, air quality is gaining increased attention in China, especially the PM_2.5_ and PM_10_ pollution. According to the air pollution source and transport rationale, the inhomogeneous air pollution distribution is mainly caused by diversity in topographic and meteorological condition, coal consumption, vehicle emissions, industrial emissions, and atmospheric transportation between southern and northern cities. In terms of the wind rose of the BTH region (shown in Figure S1), when the prevailing northerly clean wind from Inner Mongolia blows into the northern cities of the BTH region, the pollutants in the atmosphere are blown away, which lowers the exposure level. Another factor influencing exposure differences was the fact that the southern cities had received many heavy industrial companies from Beijing. Spatial comparison demonstrated that the adaptive capacity was generally lower in the northern areas than in central and southern areas. This circumstance primarily resulted from spatial differences in coal consumption and secondary industry index distribution. As for the coal consumption index diversity, cities in the first and second vulnerable classes of the coal consumption index are mainly distributed in northeastern areas; meanwhile, central and southwestern areas are mainly in the third and fourth classes. For secondary industry index distribution, southwestern cities show a higher proportion of secondary industry than northeastern cities.

The longitudinal comparisons highlight that the exposure index had an obvious downtrend from 2013 and 2015. This is mainly because, since 2012, people and the government have been more sensitive to the severe particulate air pollution that threatens human health. China’s Ministry of Environmental Protection also issued Ambient air quality standards [39] on 29 February 2012, which officially induce PM_2.5_ into normal atmospheric environment quality assessment. The Ministry of Environmental Protection also set Implementation Rules of Air Pollution Prevention Plan for the Beijing-Tianjin-Hebei region and its adjacent areas [61] in September 2013, aiming to decrease the PM_2.5_ concentration to 25% of the 2012 level by 2017. These two important decisions decreased the exposure level for most cities. Although the atmospheric environment vulnerability in the BTH region over the three years has generally improved, the atmospheric environment vulnerability is still unacceptable. This implies that decreasing air pollution is still a major long-term task for China’s government. This result is consistent with those of Jin et al. and Guo et al., who found that strenuous effort is required to improve air quality [2,62]. The longitudinal comparison also indicated that the sensitivity and adaptive capacity experienced a tiny change from 2013 to 2015.

The atmospheric environment vulnerability patterns could further impact the atmospheric environment protection management [63,64]. For example, the generally poor situation of the exposure index in the BHT region was caused by severe PM_2.5_ and PM_10_ pollution and bad AQI day indices. These exposure indices of most cities were mainly at the fourth or fifth level in the three years studied. This suggests that decision makers and managers should pay more attention to air pollution control. More specifically, all the central and southern cities need to implement comparatively strict policy limitations on air pollution emissions, such as implementing Odd–Even Day Vehicles Prohibition, shutting down highly polluting industries, and temporary suspension of kindergartens, primary and middle schools when the urban AQI is equal to or worse than grade V. These cities should concentrate on controlling PM_2.5_ and PM_10_, particularly in southwestern cities and Tangshan. Zhangjiakou, Shijiazhuang and Tangshan should improve SO_2_ pollution regulations. Shijiazhuang, Tangshan, Handan, Baoding, Xingtai, Hengshui and Langfang should reinforce NO_2_ control. Meanwhile, for the sensitivity index, the whole situation of population density and percentage of vulnerable groups is very negative in the BTH region, except for some northern cities (Zhangjiakou and Chengde). Effective and appropriate population management is an urgent task for decision makers and managers. Moreover, improving the adaptive capacity in most cities is a long-term job to decrease the general atmospheric environment vulnerability. The southern cities need to reinforce the developing of the third industry so as to accelerate economic restructuring and shift development mode. Beijing, Tianjin, Shijiazhuang, Tangshan and Baoding should be more concerned about motor vehicle population control. Tianjin, Xingtai, Baoding and Cangzhou should make more effort to strengthen the urban greening rate. The central and southern cities should increase their usage of clean energies and control coal consumption.

To provide support for atmospheric environment vulnerability management, three partitions are proposed according to the assessment results of aggregated atmospheric environment vulnerability (shown in Figure 6). The three district partitions are presented as follows: I—Districts for general control, II—Districts for key control, and III—Districts for strict control.

(1) Districts for general control. These districts consist of the regions with “potentially vulnerable” and “slightly vulnerable” vulnerability classes. These districts are mainly in the northern areas (Zhangjiakou, Chengde and Qinhuangdao), accounting for 38.75% of the area of the 13 cities in the BTH region.
(2) Districts for key control. These districts are in the vulnerability class “moderately vulnerable”, lying mainly in the central cities (Beijing, Tianjin and Cangzhou), with 16.72% of the area of the 13 cities in the BTH region.
(3) Districts for strict control. The cities with “significantly vulnerable” and “extremely vulnerable” vulnerability classes generate these districts, lying mainly in the southwestern (Baoding, Shijiazhuang, Hengshui, Xingtai, Langfang and Handan) and southeastern cities (Tangshan), accounting for 44.53% of the area of the 13 cities in the BTH region.

The developed atmospheric environment vulnerability assessment framework is demonstrated to be valid at identifying districts where natural and anthropogenic indices are accountable for current atmospheric environment vulnerability. Taking the time dimension into account makes this framework able to evaluate the influence of historical policies on atmospheric environment vulnerability and provide guidance for further atmospheric environment vulnerability management. The proposed framework allows for a comparison of atmospheric environment vulnerability if the status quo is maintained or if new decisions are introduced and allows for representation of natural and anthropogenic change under intrinsic or extrinsic stresses.

## 5. Conclusions

In conclusion, this paper evaluated the atmospheric environment vulnerability in the BTH region with spatial and temporal comparison by integrating AHP, fuzzy theory and GIS spatial analysis into an ESA framework. In the ESA framework, the logical relationships between atmospheric environment vulnerability and indices of exposure, sensitivity and adaptive capacity were elaborated upon. The vulnerability assessment effectively transforms complicated conceptual insights into operational quantification methodologies, procedures and guidance. 

The results indicate that the atmospheric environment vulnerability in the BTH region exhibits obvious spatial discrepancy, with southwestern and southeastern cities showing higher vulnerability and lower vulnerability mainly in the central and northern cities. The diverse spatial patterns indicate that exposure and adaptive capacity indices mainly influence the spatial change of atmospheric environment vulnerability and sensitivity in most cities with the same pattern, with tiny temporal and spatial differences. As the vulnerability patterns could further impact the atmospheric environment vulnerability management, managers should preferentially focus on recognized vulnerable indices. 

Future improvements of our framework should rely on better spatial databases. For decision makers in BTH regions, the policy challenge now will be to detect how to balance economic development with atmospheric environment protection in the long term.

## Figures and Tables

**Table 1:** Atmospheric environment vulnerability assessment indices of the BTH region.

Object Layer	Project Layer	Index Layer	Unit	Orientation
Integrated atmospheric environment vulnerability assessment	Exposure (E)	E1: average annual concentration of PM_2.5_	μg/m^3^	Positive
		E2: average annual concentration of PM_10_	μg/m^3^	Positive
		E3: average annual concentration of SO_2_	μg/m^3^	Positive
		E4: average annual concentration of NO_2_	μg/m^3^	Positive
		E5: Days of air quality index (AQI )equal to or better than grade II	d	Negative
		E6: Days of AQI equal to and worse than grade V	d	Positive
	Sensitivity (S)	S1: Population density	Pop. per km^2^	Positive
		S2: Percentage of vulnerable groups	%	Positive
		S3: Average annual rainfall	mm	Negative
		S4: Average annual wind speed	m/s	Negative
		S5: Average annual relative humidity	%	Positive
	Adaptive capacity (AC)	AC1: Proportion of secondary industry	%	Positive
		AC2: Motor vehicle population	million vehicles	Positive
		AC3: Coal consumption	10^3^t	Positive
		AC4: Percentage of urban greenery coverage	%	Negative

**Table 2:** AHP weights for atmospheric environment vulnerability assessment indices.

Project layer	Weight	Index Weight		
Exposure (E)	0.6145	E1: 0.2407	E2: 0.1404	E3: 0.0398
		E4: 0.0398	E5: 0.0604	E6: 0.0934
Sensitivity (S)	0.1171	S1: 0.0187	S2: 0.0308	S3: 0.0114
		S4: 0.0491	S5: 0.0071	
Adaptive Capacity (AC)	0.2684	A1: 0.0942	A2: 0.0507	
		A3: 0.0942	A4: 0.0293	

**Table 3:** The benchmark of atmospheric environment vulnerability assessment indices.

Index Layer	Atmospheric Environment Vulnerability Classes: Potential Vulnerable to Extremely Vulnerable					
	1	2	3	4	5	Source
E1: Average annual concentration of PM_2.5_	10	35	55	90	120	[41,43,44]
E2: Average annual concentration of PM_10_	20	70	100	150	180	[41,43,44]
E3: Average annual concentration of SO_2_	20	40	60	80	100	[41]
E4: Average annual concentration of NO_2_	30	40	50	60	80	[41]
E5: Days of AQI equal to or better than grade II	300	200	170	150	100	[42]
E6: Days of AQI equal to and worse than grade V	15	20	30	60	100	[42]
S1: Population density	150	300	500	700	1000	[34,45,46,47,48,49,50]
S2: Percentage of vulnerable groups	5	10	20	30	40	[34,45,46,47,48,49,50]
S3: Average annual rainfall	1000	800	500	200	100	[34,45,46,47]
S4: Average annual wind speed	3.5	2.5	2	1.5	1	[34,45,46,47]
S5: Average annual relative humidity	20	30	50	80	100	[34,45,46,47]
AC1: Proportion of secondary industry	30	38	45	55	65	[34,45,46,47,48,49,50]
AC2: Motor vehicle population	80	110	150	200	300	[34,45,46,47,48,49,50]
AC3: Coal consumption	10000	15000	20000	30000	60000	[34,45,46,47,48,49,50]
AC4: Percentage of urban greenery coverage	48	44.5	41	38	35	[34,45,46,47,48,49,50]

**Table 4:** Fuzzy membership calculation results for Beijing’s atmospheric environment vulnerability in 2015.

Indicators	Value	Membership at Different Classes					Vulnerability Rank
		A_1_	A_2_	A_3_	A_4_	A_5_	
E1	80.60	0.0000	0.0000	0.2686	0.7314	0.0000	4
E2	101.50	0.0000	0.0000	0.9700	0.0300	0.0000	3
E3	13.50	1.0000	0.0000	0.0000	0.0000	0.0000	1
E4	50.00	0.0000	0.0000	1.0000	0.0000	0.0000	3
E5	206.00	0.0600	0.9400	0.0000	0.0000	0.0000	2
E6	42.00	0.0000	0.0000	0.6000	0.4000	0.0000	3
S1	1322.63	0.0000	0.0000	0.0000	0.0000	1.0000	5
S2	20.40	0.0000	0.0000	0.9600	0.0400	0.0000	3
S3	458.60	0.0000	0.0000	0.8620	0.1380	0.0000	3
S4	2.13	0.0000	0.2600	0.7400	0.0000	0.0000	3
S5	54.92	0.0000	0.0000	0.8360	0.1640	0.0000	3
AC1	19.60	1.0000	0.0000	0.0000	0.0000	0.0000	1
AC2	561.90	0.0000	0.0000	0.0000	0.0000	1.0000	5
AC3	11,650.00	0.6700	0.3300	0.0000	0.0000	0.0000	1
AC4	48.00	1.0000	0.0000	0.0000	0.0000	0.0000	1
Exposure	-	0.0398	0.0000	0.2569	0.2176	0.0000	3
Sensitivity	-	0.0000	0.0128	0.0817	0.0040	0.0187	3
Adaptive capacity	-	0.1866	0.0311	0.0000	0.0000	0.0507	1
Atmospheric environment vulnerability	-	0.2300	0.1006	0.3783	0.2216	0.0694	3

## Supplementary Materials

The following are available online at www.mdpi.com/1660-4601/15/1/128/s1: Figure S1: The wind rose of the BTH region; Table S1: The judgement matrix and consistency ratio (CR) for atmospheric vulnerability assessment.

## Appendix A. Weights Determination

In our study, the AHP technique is applied to determine the weights of each attribute. Six steps are contained in this method, as follows:

(1) Deconstruct the decision-making problem into a hierarchical structure with “atmospheric environment vulnerability” as decision objective, which is deconstructed into the second layer with exposure index, sensitivity index, and adaptive capacity index. Each group is further deconstructed into a third layer according to its own attributes.
(2) Make decision tables for each layer of the hierarchical decomposition with pair-wise comparisons. A preference scaling approach is executed in the pair-wise comparisons with 17 scales numbers: 9, 8, 7…, 1/7, 1/8, 1/9 where 9 means the most important, 8 the second most important, and so on down to 1/9, the least important.
(3) Make a judgement matrix with the scales numbers from step 2 to establish the relationships between attributes and group index, as shown in Table S1. In the judgement matrix, 1 are the values of diagonal. If the *i_th_* row is more important than the *j_th_* column, then the value of (*i, j*) is more than 1, otherwise the *j_th_* column is more important than the *i_th_* row. Meanwhile, the value of (*j, i*) in the matrix is the reciprocal of that in (*i, j*).
(4) Determine the weights for each attribute and group index with the largest eigenvalue (*λ_max_*) of the judgement matrix as shown in Equation (A1):
  (A1) $$\lambda_{max}=\frac{\sum_{i=1}^{n}i\left[\sum_{j=1}^{n}\left(a_{ij}w_{j}\right)/w_{i}\right]}{n}.$$
(5) Estimate the consistency of the judgement matrix by consistency ratio (CR). The CR is defined as the ratio between consistency index (CI) and random index (RI), as shown in Equation (A2):
  (A2) $$CR=\frac{CI}{RI}$$
  where the consistency index (CI) is the consistency of a given evaluation matrix and calculated by Equation (A3):
  (A3) $$CI=\left(\lambda_{max}-n\right)\left(n-1\right)$$
  and the RI is a random matrix defined as the average of resulting consistency index depending on the order of the matrix. The value of CR is suggested to be less than 0.1, which means that the judgement matrix has reasonable consistency. All the CR values of each matrix in our paper are lower than 0.10, which demonstrates the suitability of the calculated weights.
(6) Aggregate the local weight of each parameter to achieve the weight of corresponding group index, as shown in Table 2.

## Appendix B. Fuzzy Theory for Index Evaluation

Fuzzy logic is one of the most effective techniques to handle uncertainties due to vague human understanding and imprecise information. Human understanding or expert opinion is best represented with degrees of certainty (fuzzy logic) rather than absolute certitude (classical logic). Zadeh (1965) introduced the fuzzy set theory in his pioneering work to deal with imprecise information. A fuzzy set is a pair *x*, *μA* (*x*):*x* ∈ *X*, in which *μA*: *x*→[0,1]. Equation (B1) represents a mathematical form of the fuzzy set theory.

(B1) F={(x,μ(x))|x∈U} ,

where *F* is the fuzzy set; *U* is the universe of discourse; *x* is any variable; and *μ* (*x*) is the membership operatory on *x* ranging from 0 to 1.

The set “*F*” is a combination of the series of subsets represented by a variable value and a membership value. Membership grades or values represent the belongingness level of a variable value on a scale of 0–1 on the linguistic scales that are more comprehensible by human intelligence. The “degree of confidence” indicates the membership grade shown in Equation (B1). Essentially, the fuzzy set theory recognizes the partial truth concept and assigns a degree of truth values between 0 and 1 to the linguistic scales.

Fuzzy synthetic evaluation based on the concept of fuzzy logic has been used in this study to develop fuzzy membership functions for each indicator. The fuzzy membership functions quantitatively model uncertainties in indicator benchmarks and inputs. Fuzzy synthetic evaluation involves fuzzification, aggregation and defuzzification to evaluate the system performance. These steps are described below.

(1) Step 1: Fuzzification

Fuzzification is a process to transfer all input values into fuzzy membership functions. In this process, defining benchmark values is the most important step. Benchmarking for atmospheric vulnerability contains indefinite, inexact, and vague information. In this study, the benchmarks have been fuzzified to handle such uncertainties. The major steps for identifying and fuzzifying the benchmarks of indicators are:

Step 1.1: Define atmospheric vulnerability indicators for each criterion under a sub-project.
A list of criteria and indicators for the project of “atmospheric vulnerability” developed in this study is presented in Table 1.
Step 1.2: Establish a common linguistic scale for developing benchmarks for each indicator.
A five-level linguistic scale was used to develop benchmarks for all the indicators based on values provided in references and expert opinion.
Step 1.3: Establish indicator values corresponding to each linguistic scale.

Indicator values are arbitrarily assigned to each of the linguistic scales based on experience and the data available in the different rating systems stated above. The benchmark of atmospheric environment vulnerability assessment indices under five scales is presented in Table 3. Table 3 illustrates the five levels of performance (or linguistic scale) with different membership functions. The triangular fuzzy functions for each linguistic scale are developed such that the most likely values (membership = 1) of one linguistic scale coincides with the minimum value (membership = 0) of next linguistic scale and the maximum value (membership = 1) of the preceding linguistic scale

(2) Step 2: Aggregation

Aggregation process synthesizes information (e.g., fuzzy sets) from the bottom of the hierarchy to the elements present at higher hierarchical levels. In this study, the aggregation process is used to develop the fuzzy sets for atmospheric vulnerability objectives by aggregating the fuzzy sets of underlying atmospheric vulnerability indicators. All indicators under each layer have been given equal importance with equal weights.

A direct assignment procedure is applied to evaluate the relative weights of atmospheric vulnerability objectives to calculate the atmospheric vulnerability at the component level. This procedure uses a linear and discrete 1–5 Likert scale represented by potential vulnerable, weakly vulnerable, moderately vulnerable, significantly vulnerable, and extremely vulnerable, respectively, and allows decision makers to prioritize objectives using simple linguistic descriptors. When one of the linguistic scales is selected, corresponding numbers on a 1–5 scale are generated. The generated numbers are normalized across all the objectives under each key component. The normalization process results in the relative weights of indicators. The fuzzy sets for atmospheric vulnerability objectives are obtained from the aggregation of related indicator fuzzy sets using the following equations:

(B2) $$F_{P}=\sum W_{I}\cdot F_{I}$$

(B3) $$F_{A}=\left[W_{I1},\;W_{I2},\;\cdots W_{In}\right]\left[\begin{array}{c} \mu_{I1}^{P}\;\mu_{I1}^{W}\;\mu_{I1}^{M}\mu_{I1}^{S}\mu_{I1}^{ES}\;\\ \mu_{I2}^{P}\;\mu_{I2}^{W}\;\mu_{I2}^{M}\mu_{I2}^{S}\mu_{I2}^{ES}\;\\ \cdots \cdots \cdots \cdots \cdots \cdots \cdots \\ \mu_{In}^{P}\mu_{In}^{W}\mu_{In}^{M}\mu_{In}^{S}\mu_{In}^{ES} \end{array}\right],$$

where *F_P_* is the sub-project corresponding to the atmospheric vulnerability objective, *F_A_* is the sub-index corresponding to the sub-project of the atmospheric vulnerability objective, and *W_In_* are the weights of the indicators, *F_I_* is the set of fuzzified values of the corresponding indicators, and $\mu_{In}^{P}\;$, $\mu_{In}^{W}\;$, $\mu_{In}^{M}\;$, $\mu_{In}^{S}\;$, $\mu_{In}^{ES}\;$ are the corresponding fuzzified values of the respective indicators.

The fuzzy sets of atmospheric vulnerability objectives are further aggregated to determine an overall atmospheric vulnerability fuzzy set, “*F_AEV_*”, for a complete atmospheric environment system:

(B4) $$F_{AEV}=\sum W_{A}\cdot F_{A},$$

where *W*_A_ are the weights of the project layers (sub-project) and *F*_A_ is the set of fuzzified values of corresponding indices under each project layer.

(3) Step 3: Defuzzification

The aggregated fuzzy sets are converted to crisp numbers using defuzzification methods in order to represent atmospheric vulnerability. In this study, to obtain crisp output of fuzzified inputs and outputs, the following centre of gravity (COG) method is used for defuzzification:

(B5) $$X_{COG}=\frac{\sum_{i=1}^{n}x_{i}\cdot \mu_{i}\left(x_{i}\right)}{\sum_{i=1}^{n}\mu_{i}\left(x_{i}\right)}.$$

In Equation (B5), the centre of gravity of the fuzzy set is located and a corresponding x-scale value is returned as a crisp number. Subsequently, the defuzzified values of atmospheric vulnerability objectives are plotted on a radar diagram.
