# Supplementary material for: Atmospheric Environment Vulnerability Cause Analysis for the Beijing-Tianjin-Hebei Metropolitan Region
Source: Int J Environ Res Public Health. 2018 Jan 13;15(1):128. doi: 10.3390/ijerph15010128 (PMC5800227; doi:10.3390/ijerph15010128)
Supplement: Supplementary file 1 [file ijerph-15-00128-s001.pdf]

Supplementary Materials: Atmospheric Environment Vulnerability Cause Analysis for the Beijing-Tianjin-Hebei Metropolitan Region

Yang Zhang, Jing Shen and Yu Li *

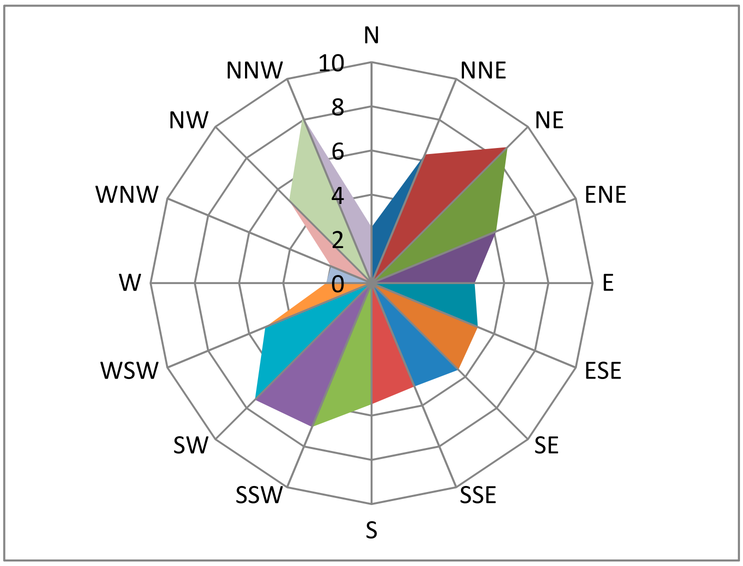

Figure S1. The wind rose of the BTH region.

Table S1. The judgement matrix and consistency ratio (CR) for atmospheric vulnerability assessment.

**Atmospheric Vulnerability Assessment**	**Exposure**	**Sensitivity**	**Adaptive Capacity**	**Weight**			
Exposure	1	4	3	0.6144			
Sensitivity	1/4	1	1/3	0.1172			
Adaptive Capacity	1/3	3	1	0.2684			
CR = 0.0707							
Exposure	E1	E2	E3	E4	E5	E6	Weight
E1	1	4	5	5	3	2	0.3916
E2	1/4	1	2	2	3	4	0.2285
E3	1/5	1/2	1	1	1/2	1/3	0.0648
E4	1/5	1/2	2	1	1/2	1/3	0.0648
E5	1/3	1/3	2	2	1	1/2	0.0983
E6	1/2	1/4	3	3	2	1	0.152
CR = 0.0816							
Sensitivity	S1	S2	S3	S4	S5	Weight	
S1	1	1/2	2	1/3	3	0.1599	
S2	2	1	3	1/2	4	0.2525	
S3	1/2	1/3	1	1/4	2	0.0973	
S4	3	2	4	1	5	0.4185	
S5	1/3	1/4	1/2	1/5	1	0.0618	
CR = 0.0152							
Adaptive Capacity	A1	A2	A3	A4	Weight		
A1	1	1	2	3	0.3509		
A2	1	1	2	3	0.3509		
A3	1/2	1/2	1	2	0.1891		
A4	1/3	1/3	1/2	1	0.1091		
CR = 0.0039							

© 2018 by the authors; licensee MDPI, Basel, Switzerland. This article is an open access article distributed under the terms and conditions of the Creative Commons by Attribution (CC-BY) license (http://creativecommons.org/licenses/by/4.0/).
